# Spatial differences in corneal electroretinogram potentials measured in rat with a contact lens electrode array

**DOI:** 10.1007/s10633-014-9459-5

**Published:** 2014-09-30

**Authors:** Yelena Krakova, Hadi Tajalli, Sanitta Thongpang, Zahra Derafshi, Tamas Ban, Safa Rahmani, Ashley N. Selner, Amani Al-Tarouti, Justin C. Williams, John R. Hetling

**Affiliations:** 1 Department of Bioengineering, University of Illinois at Chicago, 851 South Morgan Street, Rm 232, M/C 063, Chicago, IL 60607-7052 USA; 2 Department of Biomedical Visualization, University of Illinois at Chicago, 1919 W Taylor St, Chicago, IL 60612 USA; 3 Department of Biomedical Engineering, University of Wisconsin, ECB Room 3142, 1550 Engineering Drive, Madison, WI 53706 USA

**Keywords:** Electroretinogram, Rat, Topography, Spatial differences, Multi-electrode

## Abstract

**Purpose:**

It has been known for several decades that the magnitude of the corneal electroretinogram (ERG) varies with position on the eye surface, especially in the presence of focal or asymmetric stimuli or retinal lesions. However, this phenomenon has not been well-characterized using simultaneous measurements at multiple locations on the cornea. This work provides the first characterization of spatial differences in the ERG across the rat cornea.

**Methods:**

A contact lens electrode array was employed to record ERG potentials at 25 corneal locations simultaneously following brief full-field flash stimuli in normally sighted Long-Evans rats. These multi-electrode electroretinogram (meERG) responses were analyzed for spatial differences in a-wave and b-wave amplitudes and implicit times.

**Results:**

Spatially distinct ERG potentials could be recorded reliably. Comparing relative amplitudes across the corneal locations suggested a slight non-uniform distribution when using full-field, near-saturating stimuli. Amplitudes of a- and b-waves were approximately 3 % lower in the inferior quadrant than in the superior quadrant of the cornea.

**Conclusions:**

The present results comprise the start of the first normative meERG database for rat eyes and provide a basis for comparison of results from eyes with functional deficit. Robust measures of spatial differences in corneal potentials will also support optimization and validation of computational source models of the ERG. To fully utilize the information contained in the meERG data, a detailed understanding of the roles of the many determinants of local corneal potentials will eventually be required.

## Introduction

### Origins of spatially distinct corneal potentials

As the cells of the retina respond to a full-field visual stimulus, current sources and sinks form at predictable times at different distances from the vitreal surface [1, 2]. The impedances that exist between the sources and sinks result in the development of potential differences within the retina and thus electric fields. These fields can be represented as originating from an array of dipoles oriented approximately perpendicular to the vitreal surface of the retina. The spatial sum of these fields extends throughout the volume conductor of the eye and extraocular tissues, decreasing sharply with distance [3]. At the cornea, the field potentials resulting from retina-driven time-varying currents are typically between ±1 mV, depending on the stimulus, health of the retina and reference location, and are measured as the corneal ERG.

**Fig. 1:**
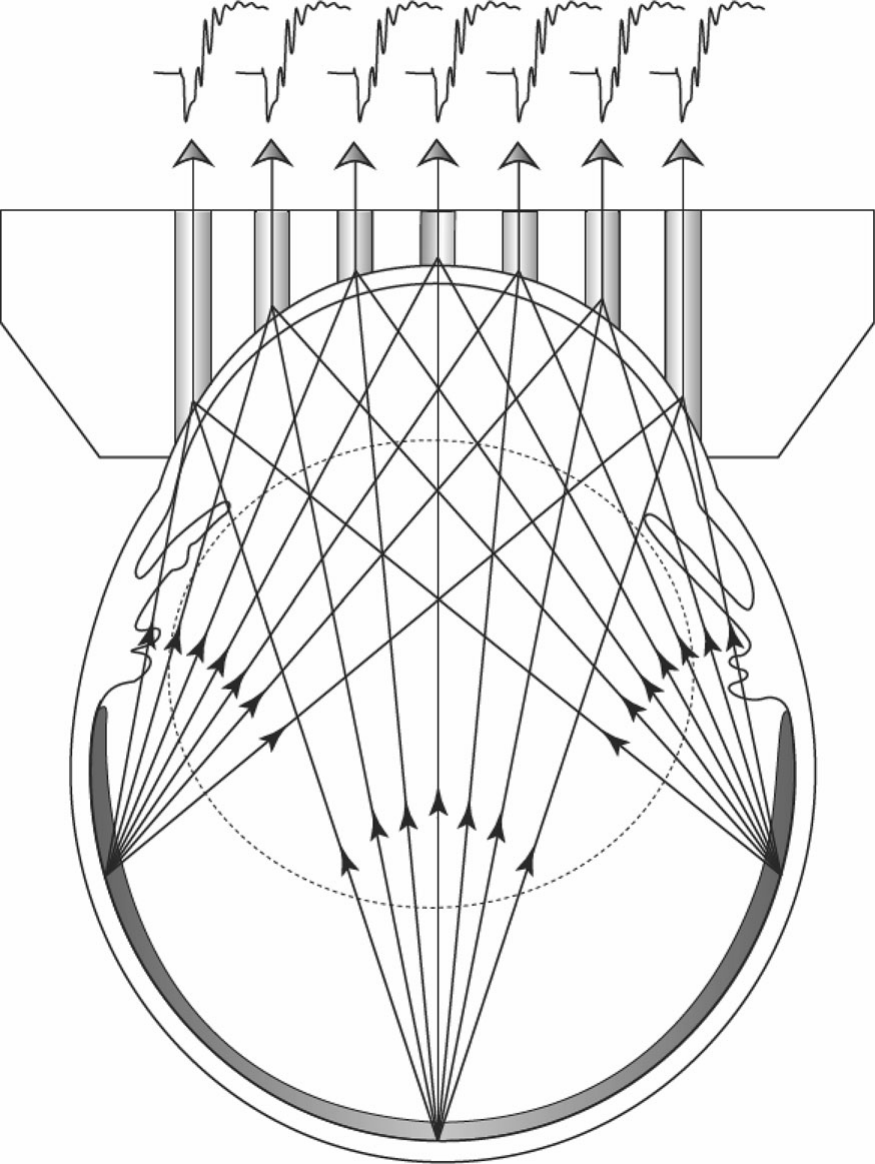
Graphical representation of multi-electrode electroretinographic (meERG) recording. Schematic cross section through a rat eye with the Contact Lens Electrode Array (CLEAr Lens) in place. *Vertical shaded* areas through the lens depict seven of the 25 saline-filled through-holes that conduct potentials from the corneal surface to the metal electrodes located on the distal planar surface of the lens. The contribution from three locations on the retina are schematized by lines radiating from each location. In use, a full-field stimulus is presented, and every responding cell in the retina contributes to the waveform recorded on each of the 25 electrodes

The full-field ERG is the sum of contributions from all of the individual cells that respond to the stimulus. At a particular point on the cornea, the contribution of an individual cell is weighted by the orientation and magnitude of its associated equivalent dipole, and the intervening impedance between that cell and the point of measurement, as represented schematically in Fig. 1.

The conductive tissues of, and surrounding, the eye create a current divider, presenting many possible paths for the retina-driven currents. When the driving dipole voltages are equal, retinal cells that are separated from the cornea by lower intervening impedance will contribute more strongly to the ERG than cells that are separated by higher intervening impedance. The intervening impedance is determined by the anatomy of the eye, the conductance of the ocular tissues, the capacitances that occur at the junctions of dissimilar resistivity, frequency of the voltage signal, and distance.

The conductance of the cornea is approximately 0.42 S/m at 100 Hz [4] and can support measureable differences in potential from one location to another in the presence of net physiological currents flowing within the cornea, parallel to the corneal surface. In the presence of a very thin tear film, the spatial differences in corneal potentials can be measured with small electrodes placed at different points of contact. This was demonstrated by Sundmark [5–7] in an extensive set of experiments using a “contact glass” containing a linear array of nine electrodes. A spatial profile of potentials was measured, but repeatability and spatial resolution were limited by poor fit of the contact glass to the subjects’ eyes and thus a thick and unstable intervening tear film. Little empirical work has been done to document spatial differences in corneal potentials since Sundmark’s contribution.

### Relevance of spatially distinct corneal potentials

Electroretinogram responses evoked by focal or multi-focal stimuli are typically recorded with electrodes that have asymmetric contact with the eye surface, such as DTL fiber electrodes (chosen because they do not alter acuity of the subject). In these approaches, the relative contribution of a given area of retina to the local corneal potential becomes more relevant. The asymmetry in corneal potentials created by asymmetric retinal activity was demonstrated by Holland and Herr [8] using rabbit eyes with laser-damage lesions. Motivated by potential bias introduced into multi-focal ERG (mfERG) recording, this issue was explored with a detailed three-dimensional numerical model of a human eye by Job et al. [9]. The modest spatial differences predicted in these studies are likely not significant and are thus not emphasized in the ISCEV standards for ERG recording other than to note that amplitudes may have to be scaled depending on where the electrode routinely contacts the eye in a given clinic or laboratory [10–12].

There have been relatively few reports on solving the forward electric field problem for the ERG (i.e., predicting eye surface potentials from assumed distributions of retinal activity) [3, 9, 13], or the inverse problem (i.e., predicting the distribution of retinal activity from measured eye surface potentials) [14, 15]. These studies have included analytical and numerical approaches, and utilized eye models of varying complexity, from a simple two-dimensional homogeneous area [20] to a three-dimensional sphere with schematized volumes representing major ocular tissues [9]. A common limitation of all of these efforts has been the availability of sufficiently robust data with which to optimize and validate the computational models. For example, the most recent numerical models of a human eye by Job et al. [9] were validated by comparison with simpler analytic models, which were in turn validated by comparison with Krakau’s [13] rabbit eye data from 1958. A significant motivation behind developing a robust multi-electrode electroretinogram (meERG) recording paradigm is to provide a deeper multi-species data set, including both normal eyes and eyes with well-defined local areas of deficit, which can be used to support future modeling efforts.

### Exploiting spatially distinct corneal potentials

If the spatial differences in corneal ERG potentials can be reliably measured, they can be interpreted to provide information about regional differences in the retinal response to the stimulus. This could be done via examination of relative differences in corneal potentials, as demonstrated by Holland and Herr [8] using rabbit eyes in vivo, and Cringle and Alder [16] using isolated perfused dog eyes; both studies compared the distribution of potentials on the ocular surface before and after creating focal laser-damage lesions. Or if coupled with a computational model of the eye, these corneal potentials might form the basis for predicting the precise location of the distributed retinal sources by solving the inverse bioelectric source problem, analogous to the electrophysiological functional mapping based on body surface potential maps demonstrated in brain [17] and heart [18]. Solving the inverse problem for the ERG has been attempted [14, 15], but efforts have been generally hindered by lack of available data with which to optimize and validate detailed electrical models of the eye. Reliable measurements of spatially distinct corneal potentials would provide this data set.

Potential benefits of a “multiple electrode + full field stimulus” approach, as compared to a “single electrode + focal stimuli” approach, are that topographical information would reflect the entire area of retina responding to the full-field stimulus (not just the area subtended by the focal stimuli), as illustrated in Fig. 1, and without stringent requirements for ocular clarity or extended fixation on a visual target during the test. It may also be possible to obtain the topography of retinal health by analysis of the response to a single brief flash.

For these reasons, a novel contact lens electrode array was designed, fabricated, and employed to record ERG potentials simultaneously from 25 locations on the cornea of rats to establish proof of concept for high-quality meERG recording. The spatial distribution of these potentials was investigated in normally sighted animals to characterize the magnitude of the spatial differences, the repeatability of the measurement, and to begin to accumulate a normative database against which to compare values obtained from animals with well-characterized retinal lesions. A guiding objective of this effort is to gain a quantitative understanding of the relationship between spatial distributions of currents within the retina and the resulting distributions of corneal potentials.

## Methods

### Animals

Long-Evans rats (purchased from Charles River, Wilmington, MA) were recorded from once within the age range 4–6 weeks, the age at which the radius of curvature of the cornea closely matched the radius of the recording lens. Animals were anesthetized with an intraperitoneal injection of ketamine and xylazine (100 and 10 mg kg^−1^, respectively), pupils were dilated with 2.5 % phenylephrine HCl and 1 % tropicamide, and the cornea anesthetized with 0.5 % proparacaine. A regulated heating pad was used to maintain animals at 35–39 °C during experiments. All experiments were in accordance with the ARVO Statement for the Use of Animals in Ophthalmic and Visual Research and approved by University of Illinois at Chicago protocol ACC 11-154.

### Contact lens electrode arrays

**Fig. 2:**
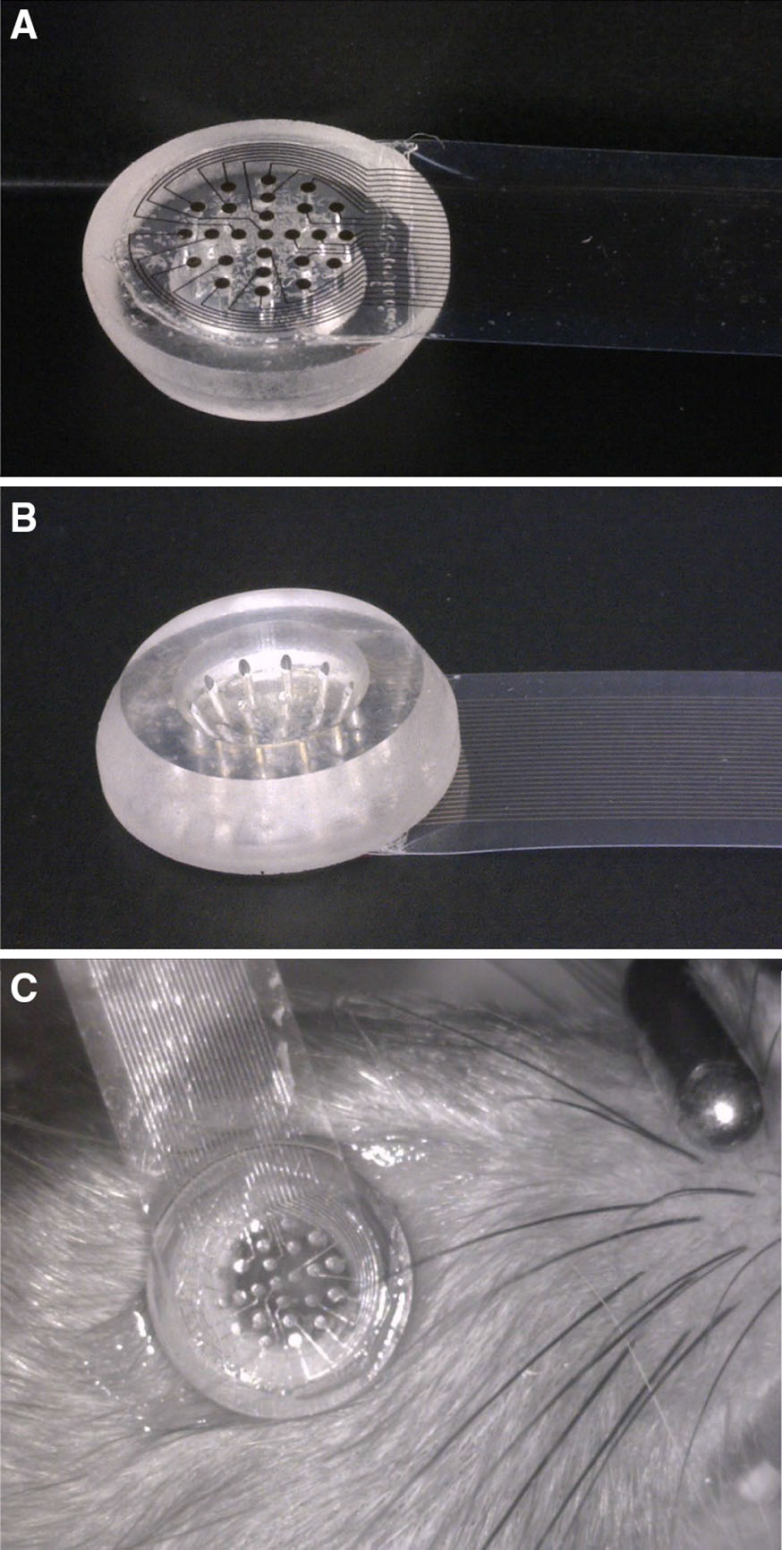
Contact Lens Electrode Array (CLEAr Lens) used for multi-electrode electroretinogram (meERG) recording from rat eyes. **a** Distal side of lens is planar and bonded to a thin-film parylene cable containing 25 electrodes. **b** Corneal side is machined to provide close contact to the cornea; optimal fit observed at 4–6 weeks of age, all recording was done within this age range. **c** In use, the cable is oriented along the superior–inferior axis; rat head is stabilized with a bite bar. Right eye shown in figure, with cable exiting in the superior quadrant; for left-eye recording, the rat is flipped, and the cable exits in the inferior quadrant

Custom contact lenses were machined from poly(methyl methacrylate) (PMMA). Each lens was concave on the corneal side and planar on the opposite surface (Fig. 2a, b). To the planar side of the lens was bonded a flexible cable; the cable incorporated exposed platinum electrodes adjacent to the lens which were joined by conductive traces to contact pads suitable for interface with a flexible printed circuit (FPC) connector at the opposite end. Clear parylene formed the cable substrate for maximum light transmission. Electrodes on the cable made electrical contact with the corneal surface via through-holes (300 μm diameter) in the lens which were filled with 1× phosphate buffered saline (Fisher Scientific) immediately prior to each recording session. Electrode impedance spectroscopy was performed on several prepared lenses; impedance of each channel was typically within the range 60–70 kΩ at 100 Hz, which was <0.1 % of the input impedance of the recording amplifier. Routine use during experiments caused no significant change in impedance or gain as verified by recording a 100 Hz sine wave immediately before and after each rat eye recording session. Due to occasional incomplete filling of the PMMA lens through-holes with saline, 22–25 out of 25 channels were typically available for each experiment.

Lens position relative to the pupil was adjusted by tension and angle of the cable until centered with the cable oriented along the superior–inferior axis as judged by eye, and later verified by measurements made on photographs taken before and after every experiment via an infrared camera installed in the stimulus source (Fig. 2c). Lens position was quite stable during recording.

The PMMA lens substrate and parylene cable are largely transparent, but the opaque metal electrodes and traces in the cable, which have a surface area of ~20 % of the area of the dilated rat pupil, do block a percentage of incident light. The percent transmission was measured to be 60–70 % (see text describing Fig. 4 below). The metal features are not near the image plane for the eye. Diffraction at the edges of the metal features, and blurring of the image, preclude distinct shadows at the retina and instead result in a distributed reduction in retinal illuminance.

### Recording

For all experiments, the stimulus was a back-lit translucent acrylic dome with a diffusing surface (i.e., frosted). The dome filled the visual field of the rat and was approximately uniform in luminance. Rats were dark adapted for at least 60 min and exposed only to dim red light during positioning of the recording lens. Platinum needle electrodes served as reference (in ipsilateral cheek) and ground (nape of neck). Stimuli consisted of brief white flashes delivered at 2-min intervals. Bright flashes were used in the present study to increase the likelihood that all areas of the retina were responding at or near-maximum levels. Flashes of either 422 or 1,842 scotopic candela seconds per square meter (sc cd s m^−2^) were delivered at least ten times in each experiment, with inter-flash intervals sufficient to maintain a dark-adapted state.

**Fig. 3:**
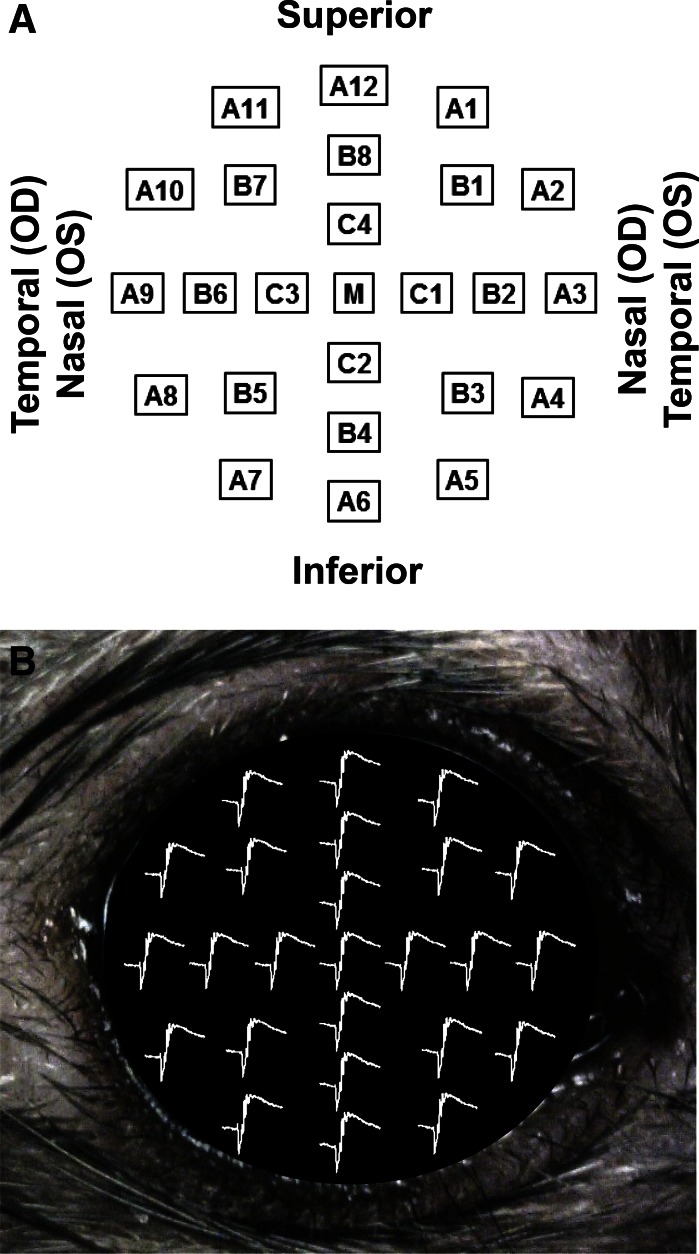
Electrode designations and anatomical orientation. **a** Electrodes are arranged in three concentric rings (*A*, *B*, *C*) plus one central electrode (*M*). Electrode A12 is always at the superior margin of the cornea. **b** Representative meERG waveforms shown in the relative positions from which they were recorded on the rat eye. The upper-most waveform was recorded by electrode A12

The Contact Lens Electrode Array (referred to below as the CLEAr Lens) contained 25 electrodes arranged in a concentric ring pattern. The electrode designations and anatomical orientation illustrated in Fig. 3 are used throughout this report. Each electrode was connected to one input channel of a commercial amplifier (MEA60, Multi-Channel Systems) modified with a custom pass-band (0.2–2,500 Hz) and interface board. Differentially recorded signals were sampled at 5 kHz and stored for later analysis.

### Analysis

A single evoked response consisted of a family of 25 ERG waveforms recorded simultaneously following a brief flash stimulus (Fig. 3b); this data set is here referred to as a multi-electrode electroretinogram (meERG), to distinguish from a conventional single-waveform ERG response. The first step in analysis of each data set was to remove waveforms that contained significant artifacts or atypically high noise levels. Corrupted meERG responses (sets of 25 simultaneously recorded waveforms) were identified by evaluating the amplitude variance at criterion pre- and post-stimulus time points across the 25 channels; high variance resulted from motion artifacts associated with blinks, twitches, or heavy breathing. Individual corrupted channels were identified by evaluating RMS noise levels in the pre-stimulus baseline; high noise levels were associated with incomplete filling of the CLEAr Lens through-holes (i.e., bubbles). After removing corrupted runs and channels, each experiment yielded 5–10 runs (repetitions of the same stimulus) and 22–24 channels per run, for further analysis.

Response a-wave amplitudes were evaluated as the excursion from the pre-stimulus baseline to the prevailing amplitude at 4 ms following the stimulus; a fixed pre-peak evaluation time was chosen over evaluating at the time of peak to minimize post-receptor contributions. Amplitudes of the b-waves were evaluated as the excursion from the a-wave trough to the b-wave peak. Implicit times for a-wave and b-wave were measured from the time of the stimulus to the time of peak.

Differences in amplitudes across the corneal surface were analyzed using a ratio approach which isolated relative differences within a single meERG response (consisting of 25 simultaneously recorded waveforms), and facilitated comparison across responses and across animals where absolute amplitude differences may be high relative to the spatial differences within one meERG response (details in Fig. 8 and associated text).

## Results

### meERG versus conventional ERG amplitudes

**Fig. 4:**
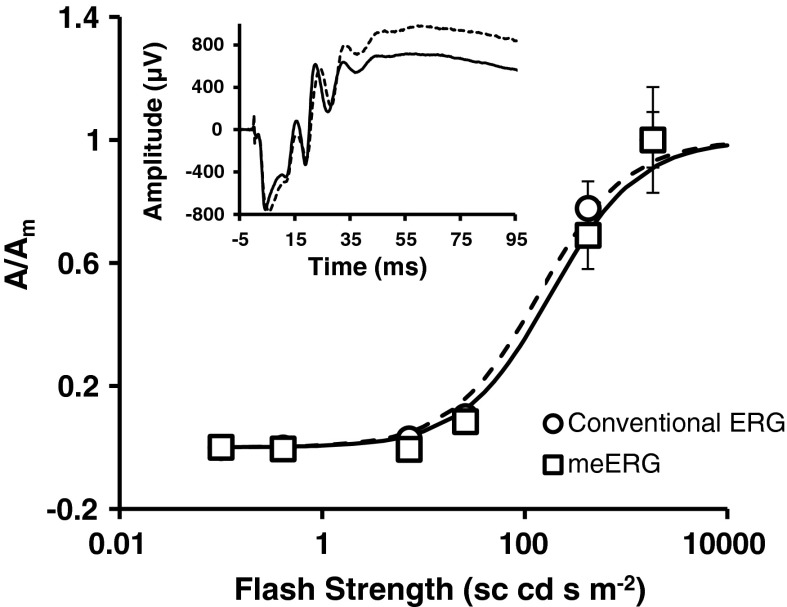
Amplitude–intensity plot comparing a-wave sensitivity for conventional (single corneal electrode) ERG with meERG responses. All data obtained in one experiment, using the same amplifier, reference, and ground electrodes. The 25 meERG waveforms recorded following each flash were averaged together, and the resulting average waveform was evaluated for a-wave amplitude. *Symbols* plot mean ± 1 SD for repeated presentations of each flash strength. Curves plot Eq. (1) fit to each data set; *dashed curve* fit to conventional ERG data (*circles*), *solid curve* fit to meERG data (*squares*). *Inset* plots a single conventional ERG response (*dashed waveform*) and a single average meERG response (*solid waveform*); flash strength = 1,842 sc cd s m^−2^ for both recording configurations

To evaluate whether meERG responses recorded with the CLEAr Lens were fundamentally equivalent to responses recorded with a conventional single-channel wire electrode, both recording methods were employed in one animal during a single experiment. For each recording technique, six flash strengths (*I*) were delivered (each repeated four times), and responses were evaluated for a-wave amplitude. Amplitudes (*A*) were normalized to the response recorded following the highest flash strength (*A*_*m*_) and are plotted in Fig. 4. Each data set was fit with Eq. (1), yielding *I*_1/2_ values of 139 and 183 sc cd s m^−2^ for conventional ERG and meERG, respectively (curves in Fig. 4). In the presence of the CLEAr Lens, 32 % more light was required to reach half-saturation, which agrees with the 60–70 % transmission measured for the lens. Response kinetics and absolute amplitudes were also similar (Fig. 4 inset).

1 $$ \frac{A}{{A_{m} }} = \frac{I}{{I + I_{\frac{1}{2}} }} $$

### Visualizing spatial differences in corneal potentials

**Fig. 5:**
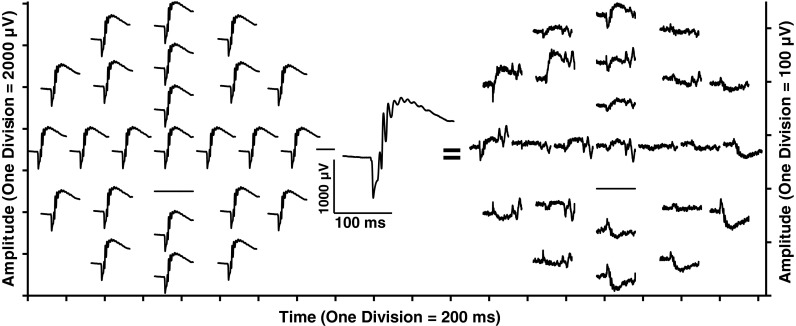
Spatially distinct ERG potentials can be measured across the cornea. meERG waveforms obtained simultaneously in response to a single flash are plotted on the left (*left vertical axis*). Waveforms are offset vertically and horizontally to appear in the relative corneal positions from which they were recorded. Note one non-functioning channel in this data set (*flat line*). These 24 waveforms were averaged together, resulting in the waveform shown in the *middle* (shown at different scale for clarity). Waveforms on the right plot the difference between each original waveform and the average of the remaining 23 waveforms; note change in amplitude scale (*right vertical axis*). If the corneal surface was isopotential, the “difference” waveforms would all be horizontal lines

A representative set of meERG response waveforms recorded from one rat is plotted in Fig. 5 (left family of waveforms). The 24 waveforms shown were recorded simultaneously in response to a single full-field flash. Each waveform is positioned according to the relative position on the cornea from which it was recorded (c.f. Fig. 3). Non-functional channels appear as flat lines (see “Methods”). Note the exceptionally large amplitudes (~2,000 μV from a-wave trough to b-wave peak) that result from the bright stimulus flash, the effective exclusion of the current-shunting tear film by the close-fitting CLEAr Lens, and the off-eye position of the reference electrode (i.e., “monopolar” recording).

To investigate whether the cornea was isopotential, a differential analysis was carried out off-line using a leave-one-out approach. Each of the 24 waveforms was referenced to the average of the other 23 waveforms, meaning that for each electrode position, the average waveform was subtracted from the original waveform at that location. The average of all 24 waveforms is shown in the center of Fig. 5, enlarged to show detail. The resulting “difference” waveforms are plotted at the right side of Fig. 5 (note different scale). Deviations of the difference waveforms from zero (flat line) illustrate that the corneal electrodes in the CLEAr Lens are not electrically “shunted” through the tear film, and that the local corneal potentials are not identical, even for a healthy rat responding to a full-field stimulus.

**Fig. 6:**
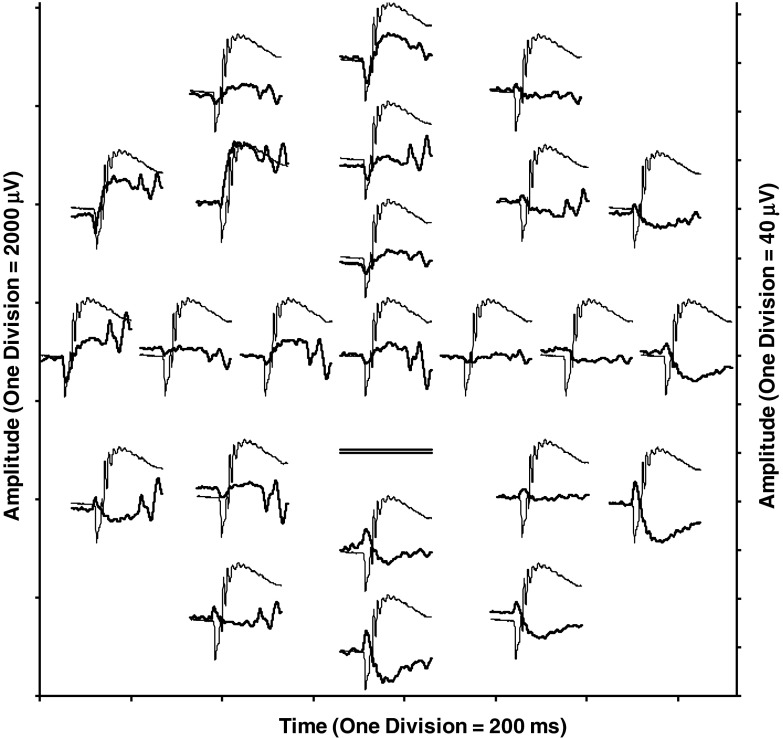
Original meERG waveforms (*thin traces*, *left amplitude axis*) and difference waveforms (*thick traces*, *right amplitude axis*) from Fig. 5 overlaid to clarify variation of a-wave and b-wave amplitudes across the corneal surface. Where original waveform was larger than the average, the a- and b-wave polarities are preserved in the difference waveforms (e.g., electrodes A9–A12 in the temporal and superior quadrants). Where original waveform was smaller than the average, the a- and b-wave polarities are inverted in the difference waveforms (e.g., electrodes A2–A7 in the nasal and inferior quadrants)

To visualize trends in the spatial differences of meERG response waveforms, Fig. 6 plots the original and difference waveforms from Fig. 5 overlaid at the same coordinates for each electrode position. For this figure only (not Fig. 5), the difference waveforms have been smoothed with a ± 1.5 ms moving average filter. Looking at the responses recorded by the peripheral electrodes, the original waveforms in the temporal and superior quadrants (A9–A12; refer to Fig. 3) were larger than the average of the remaining waveforms, and so, the polarity of the a- and b-waves in the difference waveforms is preserved. For electrodes in the nasal and inferior quadrants (A2–A7), the original waveforms were smaller than the average of the remaining waveforms, and so, the difference waveforms appear inverted, with a positive deflection corresponding to the a-wave and a negative deflection corresponding to the b-wave.

**Fig. 7:**
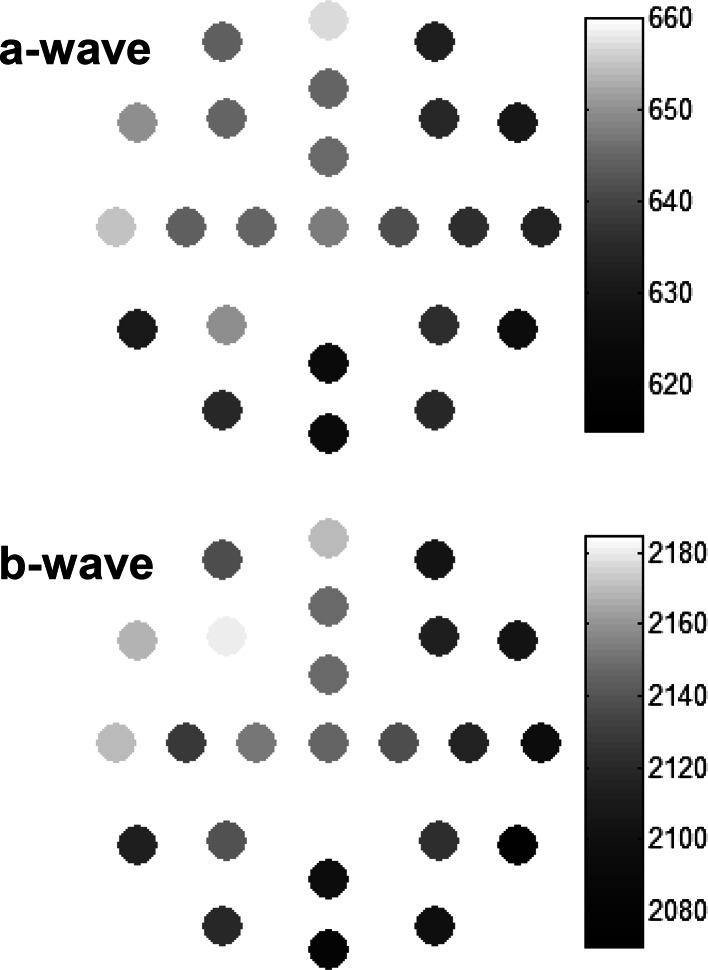
Topography of corneal potentials for one representative animal, right eye. *Grayscale* *coding* indicates the absolute amplitude of the meERG response at each CLEAr Lens electrode location; numbers are in μV. *Upper panel* plots a-wave amplitudes; *lower panel* plots b-wave amplitudes. The a-wave and b-wave amplitudes tend to be smaller (*darker circles*) in the nasal and inferior quadrants. Location with no value plotted was an electrode channel removed from analysis (*C2*, see “Methods”). Electrode layout and anatomical orientation as in Fig. 3. Max and min values for grayscale coding were scaled to highlight relative differences within each panel

To further aid visualization of the relative amplitude differences, the meERG amplitudes observed in a representative animal are plotted in Fig. 7 using a grayscale coding. The absolute a-wave and b-wave amplitudes (i.e., magnitudes) are plotted in the upper and lower panels, respectively, for the response to a single flash. The electrodes in the inferior/nasal cornea (lower right side of each plot) recorded, on average, lower amplitudes (darker circles) than electrodes in the superior/temporal hemisphere (upper left side of each plot). The maximum and minimum values of local a- and b-wave amplitudes are tens of microvolts apart in each panel, corresponding to local minima that are 4–6 % below the local maximum. Relative differences across electrode locations are highly correlated from a-wave to b-wave.

### Quantifying spatial differences in corneal potentials

The differential amplifiers universally employed in electroretinography remove much, but not all, of the noise from the recorded signals. The remaining noise originates in the animal or in the recording system. In meERG recording, noise originating in the animal will be highly correlated across the 25 channels, and averaging simultaneously recorded waveforms across channels does not reduce this noise contribution. The typical baseline RMS value for the original meERG waveforms in Fig. 5 (left) is 11.2 μV; the baseline RMS value for the averaged waveform shown in the center of Fig. 5 is 10.7 μV. The difference in squared RMS values [(11.2 μV)^2^–(10.7 μV)^2^] represents an estimate of the noise power not correlated across the channels, and is approximately 9 % of the total noise in the “raw” waveforms. The typical baseline RMS value for the difference waveforms in Fig. 5 (right) was 1.7 μV; this also is an estimate of the noise not correlated across the channels [(1.7 μV)^2^] and is approximately 2 % of the noise power in the “raw” waveforms. Therefore, approximately 2–9 % of the noise in the present meERG data is inherent in the recording system. The remainder is highly correlated across channels and originates primarily from non-ERG physiological activity or movement due to breathing and heartbeat which is sensed differently by the CLEAr Lens and the reference electrode. This correlated noise (approximately 95 % of the noise in the raw waveforms) can be removed by evaluating ratios between different electrode locations rather than absolute amplitudes.

**Fig. 8:**
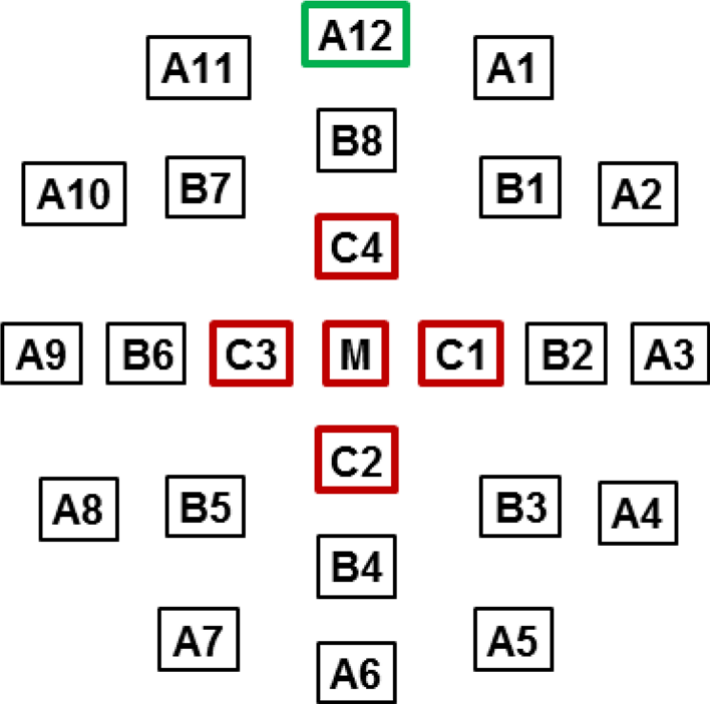
Strategy for calculating ratios used to evaluate spatial symmetry in corneal potentials. Waveforms at each position were first evaluated for amplitude or implicit time. The value at each peripheral electrode (*A1*, *A2*, *A3*,… *A12*, *green box*) was then divided by the mean value obtained from the central five electrodes (*C1*, *C2*, *C3*, *C4*, *M*, *red boxes*). This procedure resulted in twelve ratios representing the relative amplitudes along twelve radial directions from the corneal pole, each direction separated by 30°

Ratios can also be used as a normalization technique, to facilitate comparison of small spatial differences across data sets. Compared to all other available ERG recording techniques, the novel information provided by meERG recording is the spatial differences in corneal potentials. A central question was whether the spatial differences as shown in Figs. 5, 6, 7 would be consistent across animals. Figure 8 illustrates the strategy chosen, where the potential measured on each peripheral electrode (A1–A12) was divided by the mean of the potentials recorded on the central five electrodes (C1–C4, M), yielding twelve ratios. This approach removes the influence of fluctuations in absolute amplitude, isolates spatial differences in corneal potentials, and facilitates pooling data across responses within an experiment and across separate experiments. Ratio analysis has been applied to mfERG data for similar reasons [19, 20].

**Fig. 9:**
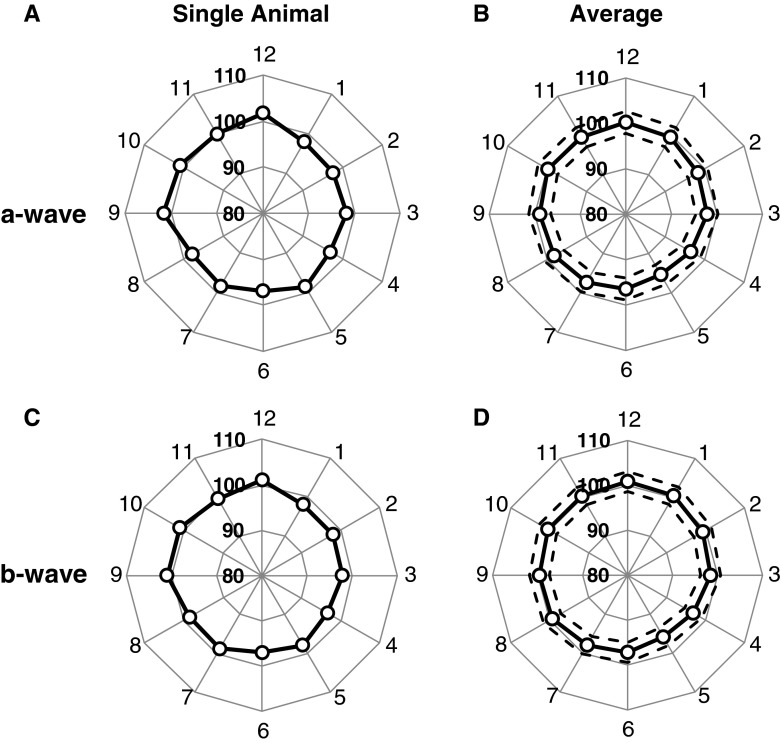
Radar plots used to evaluate radial symmetry in meERG amplitudes. *Numbers* around the perimeter of the plot correspond to the A-ring electrodes (*A1*, *A2*,…, *A12*). Distance from the origin is the ratio (c.f. Fig. 8), expressed as a percentage. **a** *Symbols* plot ratios calculated for a-wave amplitudes in a single animal. **b** *Symbols* plot mean a-wave ratios obtained in eight animals; *dashed lines* plot ±1 SD. **c** *Symbols* plot ratios calculated for b-wave amplitudes in a single animal. **d** *Symbols* plot mean b-wave ratios across eight animals; *dashed lines* plot ±1 SD. Flash strength = 1,842 sc cd s m^−2^

For each animal, the meERG waveforms were evaluated for a-wave and b-wave amplitude, and these amplitudes were averaged over the number of repeated stimuli of the same strength within that experiment (typically 5–10). Using the averaged amplitudes for each animal, ratios were calculated for each of the 12 peripheral electrode positions (c.f. Fig. 8) and multiplied by 100. Examples of the a-wave and b-wave amplitude ratios for an individual animal are plotted in the radar plots of Fig. 9a, c where the ratio for each of the 12 electrode positions is plotted as the distance from the origin in the direction corresponding to each electrode. The 12 ratios obtained from each of eight animals (right eyes, flash strength = 1,842 sc cd s m^−2^) were averaged across animals for each electrode position; these mean ratios are plotted in Fig. 9b, d. The standard deviation (SD) was calculated for each electrode position (across animals) and averaged across the 12 electrode positions; ±1 SD is indicated by the *dashed lines*. Ratios calculated for inferior electrode positions (A5, A6, A7) were slightly, yet consistently, lower than ratios observed for the superior electrode positions (A11, A12, A1). Comparing these subsets of electrode positions yielded *p* < 0.001 (Student’s unpaired *t* test). A similar comparison of nasal (A2, A3, A4) versus temporal (A8, A9, A10) electrode positions revealed marginal asymmetry (*p* = 0.025), suggesting that the true axis of asymmetry was not precisely aligned with the superior–inferior axis.

**Fig. 10:**
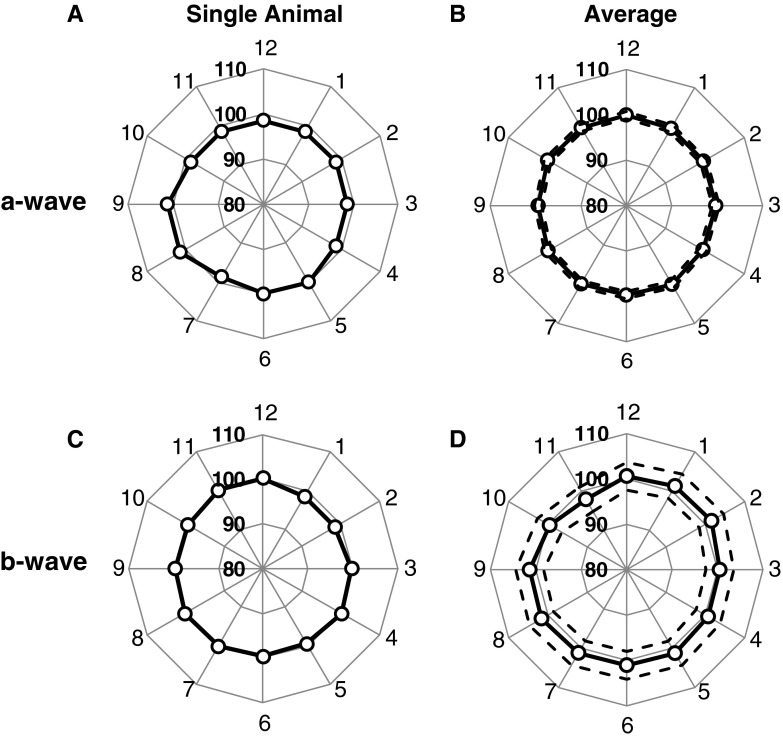
Radar plots used to evaluate radial symmetry in meERG implicit times. Plots formatted as in Fig. 9; data from same experiments as in Fig. 9. **a** *Symbols* plot ratios calculated for a-wave implicit times in a single animal. **b** *Symbols* plot mean implicit time ratios obtained in eight animals; *dashed lines* plot ±1 SD. **c** *Symbols* plot ratios calculated for b-wave implicit times in a single animal. **d** *Symbols* plot mean b-wave implicit times across eight animals; *dashed lines* plot ±1 SD

An approach similar to that in Fig. 9 was used to evaluate spatial differences in implicit time for the a-wave and b-wave; these results are plotted in Fig. 10. The implicit times recorded by peripheral electrodes were more uniform relative to the central electrodes, with no significant asymmetry along superior–inferior or nasal–temporal axes.

**Fig. 11:**
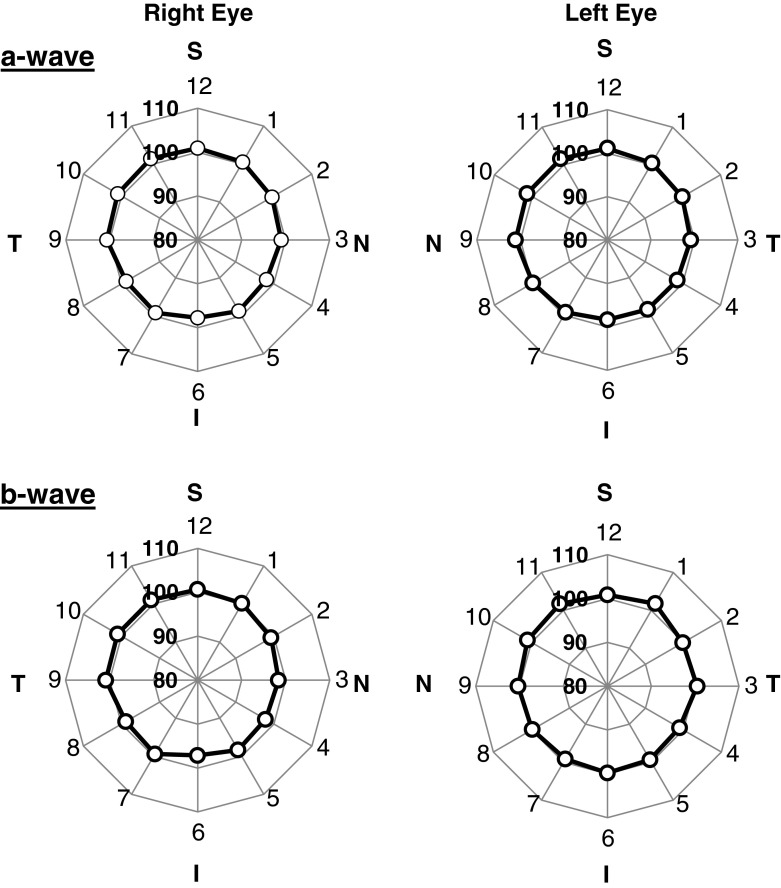
Comparison of radial symmetry in left and right eyes. Results from two representative animals (one right eye, one left eye); plots arranged and oriented as if viewing the animal from the front. *S*, *N*, *I*, *T* indicate superior, nasal, inferior, and temporal, respectively. *Numbers* around the perimeter of the plot correspond to the A-ring electrodes (*A1*, *A2*,…, *A12*). Distance from the origin is the ratio (c.f. Fig. 8), expressed as a percentage. *Upper panels* plot a-wave amplitude ratios; *lower panels* plot b-wave amplitude ratios. Flash strength = 422 sc cd s m^−2^

**Fig. 12:**
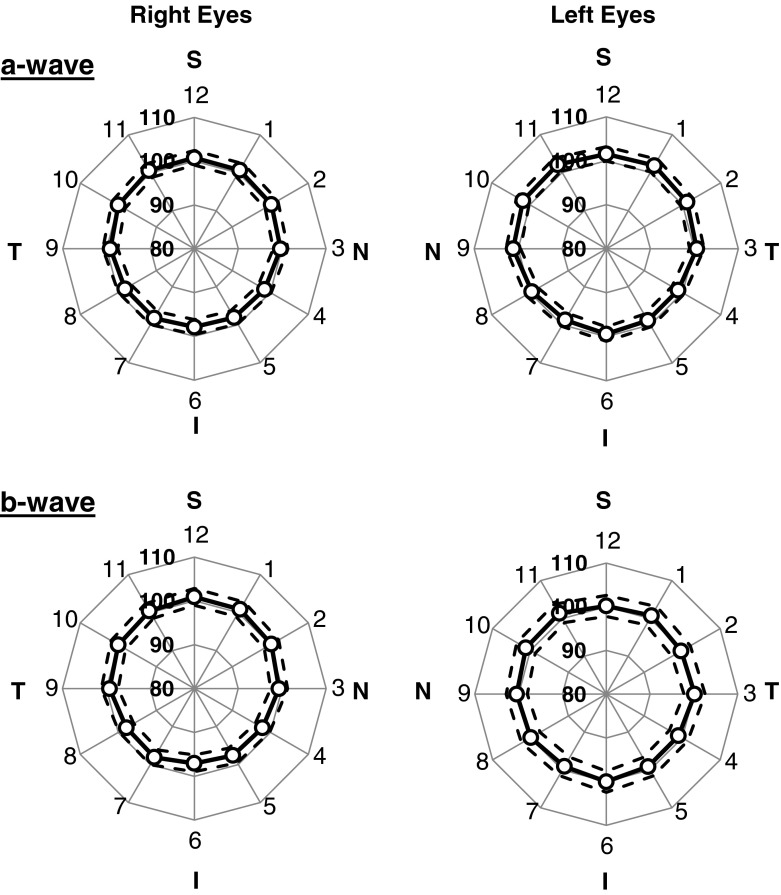
Comparison of radial symmetry in left and right eyes. Results from seven right eyes and five left eyes, including the two animals in Fig. 11. Plots arranged as in Fig. 11. *Symbols* plot mean a-wave ratios (*upper panels*) and b-wave ratios (*lower panels*); *dashed lines* plot ±1 SD. Degree of radial symmetry is quantified in Table 1

A second group of 12 animals was used to compare meERG responses from right eyes (*n* = 7) to left eyes (*n* = 5). The protocol was similar to that above, but used a flash strength of 422 sc cd s m^−2^. For right and left-eye recording, the CLEAr Lens cable exited at different quadrants of the eye (see Fig. 2 legend). This allowed analysis of whether the superior–inferior asymmetry followed the eye or the CLEAr Lens orientation. Results from two representative eyes are plotted in Fig. 11, and the mean responses from all 12 eyes are plotted in Fig. 12. Amplitude ratios obtained in each quadrant (superior, nasal, inferior, temporal; S, N, I, T, respectively) were compared along the S–I and N–T axes; the results appear in Table 1. For a-wave amplitudes, there is a small but significant S–I asymmetry but no such difference along the N–T axis. The bias of this asymmetry was toward higher ratios observed in the superior quadrant, even when the CLEAr Lens was rotated 180° between right-eye and left-eye recording. The S–I asymmetry was also seen for b-wave amplitudes in the right eyes, but not in the left eyes. Ratio values for b-waves showed no significant asymmetry along the N–T axis.

**Table 1:** Asymmetry in meERG amplitude ratios in left and right eyes

	A-wave	B-wave
Right eyes (*n* = 7)		
Superior (11, 12, 1)	100.7 ± 1.7	100.7 ± 1.8
Inferior (5, 6, 7)	98.1 ± 1.9	97.6 ± 2
*p*	<0.0001	<0.0001
Nasal (2, 3, 4)	99.5 ± 1.7	99.2 ± 2
Temporal (8, 9, 10)	99.1 ± 1.7	99.0 ± 1.9
*p*	0.5	0.8
Left eyes (*n* = 5)		
Superior (11, 12, 1)	101.8 ± 1.7	100.7 ± 2.9
Inferior (5, 6, 7)	99.0 ± 1.6	99.4 ± 2.4
*p*	<0.0001	0.2
Nasal (2, 3, 4)	100.8 ± 2	100.5 ± 2
Temporal (8, 9, 10)	100.2 ± 1.8	99.7 ± 1.9
*p*	0.4	0.3

*p* values obtained using Student’s unpaired *t* test, comparing the groups of ratio values obtained in each experiment for the electrode channels indicated in the first column

To assess the direction of the axis of asymmetry, the twelve mean ratio values in each panel of Fig. 12 were taken as vectors, which were then summed to yield an equivalent vector. For a-wave ratios, this vector pointed 6 degrees nasal of superior for right eyes and 10° nasal of superior for left eyes. For b-wave ratios, the equivalent vector pointed 2° nasal of superior for right eyes and 30° nasal of superior for left eyes. Thus, the directional bias of the axis of asymmetry was consistent with the anatomy (always slightly nasal), even though the lens was rotated 180° between right and left-eye recording.

To further ensure that small differences in gain between CLEAr Lens channels were not responsible for observed differences in corneal potentials, a sine wave was introduced to all channels in the lens simultaneously at the start and conclusion of every experiment. This was achieved by inverting the lens, filling the lens cup with saline, and contacting the saline with a wire from the sine wave source. Amplitude ratios were calculated and analyzed as described above, and the resulting values are given in Table 2. While small differences in sine wave amplitude were observed across the channels, there was no systematic asymmetry along the S–I or N–T axes.

**Table 2:** Asymmetry in sine wave amplitude ratios used to evaluate uniformity of gain across the 25 CLEAr Lens channels

	Pre experiment	Post-experiment
Sine wave amplitude ratios (*n* = 12)		
Superior (11, 12, 1)	100.5 ± 0.3	100.5 ± 0.3
Inferior (5, 6, 7)	100.2 ± 0.7	100.2 ± 0.7
*p*	0.1	0.1
Nasal (2, 3, 4)	100.1 ± 0.5	100.1 ± 0.5
Temporal (8, 9, 10)	100.5 ± 0.6	100.5 ± 0.7
*p*	0.1	0.1

*p* values obtained using Student’s unpaired *t* test, comparing the groups of ratio values obtained in each experiment for the electrode channels indicated in the first column

## Discussion

To the authors’ knowledge, this is the first attempt to measure the topographical distribution of corneal ERG potentials since Sundmark’s work over 50 years ago [5–7]. The present study was motivated by the potential clinical relevance of the information gained via multi-electrode ERG (meERG) recording, specifically related to (1) focal and multi-focal stimuli protocols that may be biased by location of electrode contact with the eye surface, (2) the relationship between the spatial distribution of corneal potentials with the spatial distribution of retinal activity, and (3) the potential for using meERG data to optimize and validate bioelectric models of the eye toward electrophysiological functional imaging of the retina.

### Impact on single-electrode ERG recording

The increasing number of studies using mfERG was a primary motivation behind the modeling work reported by Job et al. [9]. That report examined the effect of focal stimulation and focal lesions on the distribution of corneal potentials in human eyes, finding spatial differences on the order of 1 % for healthy human eyes receiving full-field stimulation, but as much as 10 % for focal stimulation of peripheral retina. In typical ERG recording, however, the cornea is covered by a layer of low-impedance tears (natural or artificial, typical conductivity ~1.50 S/m). The presence of the high-conductance tear film during ERG protocols utilizing DTL, gold foil, or wick-type electrodes likely shunts the potential differences over the corneal surface, and minimizes the effect of local recording bias. For full-field ERG protocols using ERG recording electrodes designed to have a substantial area of electrical contact with the eye and tear film, via metal rings (Burian Allen, JET, Doran Gold Lens, HK loop), the electrodes effectively average the potentials over the area of contact. For these reasons (spatial shunting via tear film, spatial averaging via large contact area), spatial differences in corneal potentials can be largely ignored in routine ERG recording.

### Clinical relevance

The obvious clinical application of meERG recording is analysis of the spatial differences in corneal potentials to detect regional deficits in retinal health. A *qualitative* relationship between local laser-damage lesions at the retina and resulting changes in the distribution of potentials on the eye surface has been demonstrated in rabbit and dog eyes [8, 16]. Determining the *quantitative* relationship between the distributions of retinal sources and corneal potentials will require a repeatable means of recording those potentials at a reasonable spatial sampling, as demonstrated here with the CLEAr Lens.

Variance in amplitudes across repeated stimuli and across subjects results in broad distributions in normative data for conventional ERG recording. By evaluating relative differences as a function of location on the cornea using a ratio approach (c.f. Figs. 8, 9, 10, 11, 12), variability in absolute amplitude is removed from the analysis. Spatial differences in corneal potentials are a source of information derived from meERG recording that was not previously available and are shown here to be repeatable, with typical SDs of 2–3 % across animals and 1–2 % across repeated stimuli within an animal. An index derived from locally measured corneal potentials may eventually provide a sensitive indicator of regional deficits in retinal function, such as associated with early-stage progressive eye disease.

The main results of the present work were (1) to demonstrate the feasibility of meERG recording using an appropriately designed contact lens electrode array (CLEAr Lens) and (2) to begin to characterize the normative meERG response in rat. Data from the first 30+ experiments with rats performed in our lab are not included in this initial report because progressive refinements to the meERG infrastructure and protocols resulted in progressively lower response variance between repeated stimuli and between animals (a trend that continues). A highly repeatable response was recorded in the 20 consecutive experiments reported here. These data represent the beginning of a normative meERG database for pigmented rat, to be used for future comparison with meERG data obtained from eyes with known regional functional deficit. To fully utilize the information contained in the meERG data, a detailed understanding of the roles of the many determinants of local corneal potentials will eventually be required.

### Sources of asymmetric corneal potentials

There was no a priori expectation of spatial uniformity or non-uniformity in the meERG potentials; however, a radial symmetry was expected based on the approximately radial symmetry of the ocular anatomy and cell distribution across the retina. The results above indicate a modest, yet repeatable, superior–inferior asymmetry in the corneal potentials. Possible reasons for this asymmetry include inhomogeneous distribution of the stimulus energy across the retina, inhomogeneous responsivity of the retina (due to cell density, photoreceptor orientation, outer segment length [21]), asymmetry in the conductivity or anatomy of the ocular tissues, or non-uniform effective gain applied to each electrode. Introducing a sine wave to all CLEAr Lens electrodes simultaneously revealed small effective gain differences, most likely due to small variations in electrode impedance, which could explain, at most, only 10–12 % of the superior–inferior asymmetry noted for a-wave and b-wave amplitudes (i.e., 10–12 % of the observed 3 % differences given in Table 1).

A careful evaluation of the effect of the CLEAr Lens on the spatial distribution of the stimulus energy at the retina has not been carried out. To the extent possible by visual examination via an infrared camera installed in the stimulus source, the rat eye was routinely positioned so that it was centered below the concave, hemispheric light source, with the optical axis of the eye aligned with the apex of the hemisphere; the CLEAr Lens was positioned such that the planar surface was normal to the optical axis of the eye. Slight misalignment could produce asymmetry in the retinal illuminance, but examining photographs taken of the rat eye before and after each experiment did not reveal any systematic error. The parylene cable of the CLEAr Lens contains metal traces that are not radially symmetric, with the primary asymmetry along the superior–inferior axis (Fig. 2a, c). However, the asymmetry observed in corneal potentials was independent of the lens orientation, which was rotated 180° between left and right eyes. Further, the responses analyzed for the present study were all evoked with stimuli that were at or near saturation, so that for a modest inhomogeneity in retinal illuminance, even areas of relatively low illumination would respond at near-maximum amplitudes.

The asymmetric corneal potentials observed in the present study could reflect asymmetric distributions of photoreceptors. The bright-flash-evoked responses recorded under dark-adapted conditions contain contributions from both rod and cone pathways. The meERG responses were evaluated for a-wave and b-wave amplitude, reflecting primarily rod photoreceptor and ON-bipolar cell activity, respectively. To the authors’ knowledge, there has not been a report of the topography of rod photoreceptor density across the entire rat retina. However, a number of reports have documented retinal ganglion cell (RGC) density across the entire rat retina, revealing distinct radial and circumferential asymmetry that is either repeatable [22, 23] or highly variable [24] between animals. The latter reports find the highest RGC density in the superior–temporal quadrant. Complete cone density maps have also been created, with the observation that L-cone distributions are positively correlated with RGC distributions and negatively correlated with S-cone distributions in pigmented rats [23]. A detailed histological analysis of individual animals following meERG recording would be interesting, but may be academic as inter-animal or inter-subject variability will define the measurable effect size regardless of the source of the variability.

### Multi-electrode ERG versus multi-focal ERG

A quantitative comparison between mfERG and meERG is not appropriate at this early stage of meERG development; however, a qualitative comparison may help place meERG recording in context. In a typical mfERG recording protocol, a dynamic patterned stimulus is presented via a video monitor for 4–8 min per eye, and the response of the retina is recorded continuously during the test using a single recording electrode [25]. This single continuous waveform is analyzed to correlate temporal voltage changes in the recorded signal with spatio-temporal luminance changes in the stimulus; these correlated voltage changes are then assigned to the area of the retina that corresponds to that location in the stimulus image [26]. Given the typically high frame frequency for the mfERG stimulus (60–75 Hz), all areas of the retina subtended by the stimulus pattern, which typically covers the central 40°–50° of visual angle, contribute to the recorded signal at every point in time. mfERG recording enables unprecedented spatial resolution in the local luminance response of the central retina, and a great deal of effort over the past 20 years has been devoted to relating the mfERG response waveforms with the bioelectric events of the retina [25]. Challenges to mfERG recording include test durations that are difficult for some subjects, a requirement for good acuity and ability to fixate on a target during the test, and technical difficulty in probing peripheral retina and isolating rod pathways.

In contrast, meERG uses a full-field (Ganzfeld) stimulus, and is therefore compatible with any full-field stimulus protocol (e.g., scotopic, photopic, chromatic, flicker, paired-flash, step or “sawtooth” stimuli), and has no stringent requirement for fixation or ocular clarity. The high luminance available with standard Ganzfeld sources plus the stable contact of the CLEAr Lens afforded excellent signal to noise ratio in the present work; for any given ERG protocol, higher SNR translates to shorter test times by reducing the need to average responses. As the Ganzfeld source illuminates the entire anatomical retina, the meERG response reflects the entire retina; however, it is important to understand that the corneal electrode array does not “map” directly to individual retinal areas but is rather coupled through a weighting matrix that relates every part of the retina to each electrode (Fig. 1). In meERG recording, the topographical luminance response information is obtained by analyzing differences in *space* (location on the cornea), not in *time* as in mfERG. Therefore, in theory, all of the topographical information available could be gained in the response to a single flash. The cellular sources of the meERG response are also relatively simple to interpret, being no different from conventional single-electrode ERG waveforms. More time and effort will be required to evaluate the spatial resolution and sensitivity of meERG recording to local areas of deficit of the retina; however, it is unlikely that the spatial resolution of such analysis will ever match the mfERG due to the “blurring” of the local retinal contributions at the cornea. With further development, we anticipate that the low-resolution, whole-retina information gained via meERG recording may be complimentary to the high-resolution, central-retina information provided by mfERG in clinical and research settings.
